# Efficacy of the Buzzy® device for pain management of children during needle-related procedures: a systematic review protocol

**DOI:** 10.1186/s13643-018-0738-1

**Published:** 2018-05-22

**Authors:** Ariane Ballard, Christelle Khadra, Samara Adler, Evelyne Doyon-Trottier, Sylvie Le May

**Affiliations:** 1 Faculty of Nursing, University of Montreal, 2375, Chemin de la Côte-Ste-Catherine, Montreal, QC H3T 1A8 Canada; 2 CHU Sainte-Justine Research Centre, 3175, Chemin de la Côte-Ste-Catherine, Montreal, QC H3T 1C4 Canada; 3 Faculty of Medicine, University of Montreal, 2900, boulevard Édouard-Monpetit, Montreal, QC H3T 1J4 Canada; 4 Division of Emergency Medicine, Department of Pediatrics, CHU Sainte-Justine, 3175, Chemin de la Côte-Ste-Catherine, Montreal, QC H3T 1C4 Canada; 5 Faculty of Nursing, University of Montreal, P.O. Box 6128, Succursale Centre-Ville, Montreal, QC H3C 3J7 Canada

**Keywords:** Pediatric, Children, Pain management, Needle-related procedures, Buzzy, Cold, Vibration

## Abstract

**Background:**

Needle-related procedures are the most important source of pain in children in hospital setting. Unmanaged pain could result in short- and long-term physiological, psychological, and emotional consequences. Although the efficacy of numerous interventions has been evaluated, procedural pain management is often suboptimal in children undergoing needle-related procedures. The main objective of this systematic review is to examine the evidence for the efficacy of the Buzzy® device for needle-related procedural pain in children.

**Methods:**

An electronic search will be conducted in the following databases: CENTRAL, PubMed, MEDLINE, EMBASE, PsycInfo, and CINAHL. There will be no restriction regarding the language, date of publication, and publication status. Eligible studies will be randomized controlled trials using the Buzzy® device for pain management in children undergoing needle-related procedures. Selection of studies, data extraction and management, assessment of risk of bias and quality of evidence will be performed by two independent reviewers. A third researcher will be consulted in case of discrepancies. Depending on the availability and quality of the data as well as clinical and statistical heterogeneity, a meta-analysis will be performed. Otherwise, findings will be qualitatively reported.

**Discussion:**

This will be the first systematic review to examine the efficacy of the Buzzy® device on pain management of children during needle-related procedures. Results of this review will guide clinical practice and recommendations for further research to improve procedural pediatric pain management.

**Systematic review registration:**

PROSPERO CRD42017076531

**Electronic supplementary material:**

The online version of this article (10.1186/s13643-018-0738-1) contains supplementary material, which is available to authorized users.

## Background

### Description of the problem or issue

Needle-related procedures, such as venipuncture and intravenous cannula insertion, are considered as the most important par sources of pain and distress in children in different settings [1–4]. Pain management during these procedures is of outmost importance as pain could result in numerous physiological, psychological, and emotional consequences [5–7]. A severe consequence of unmanaged procedural pain in children is needle phobia, defined as an extreme fear and anxiety associated with needles [8–10], which usually develops between the ages of 5 and 10 following a bad needle experience [11, 12]. Indeed, a cross-sectional survey showed that more than 60% of children reported a fear of needles [9]. In short term, the fear can generate serious physiological symptoms during needle-related procedures, such as hypoxemia [13, 14], vasovagal reactions [15–17], tachycardia, and change in hormone levels [15, 17]. Children with needle phobia are also at risk of presenting fear of healthcare professionals and experience as well as higher levels of pain and fear during subsequent procedures [17–19]. Long-term consequences include the avoidance of the healthcare system and of procedures requiring needles (e.g., blood test) [9, 10, 15] and the non-compliance with vaccination and other health conditions [15, 20].

To date, numerous pharmacological, physical, and psychological interventions have been evaluated for pain management in children undergoing needle-related procedures, such as distraction and topical anesthetic. Most of these interventions required short training for healthcare professionals and can be perceived as being time consuming and/or expensive causing barriers to implementation in the clinical settings and daily practice [1, 21, 22]. Nonetheless, an easy-to-use, rapid, non-invasive, inexpensive, and reusable intervention could be an interesting alternative, particularly in acute settings, which are often busy and where time to perform a procedure is an issue.

### Description of the intervention

Buzzy® (MMJ Labs, Atlanta, GE, USA) was created by a pediatrician and a nurse looking for an easy-to-use, reusable, and rapid intervention for pain management of children undergoing needle-related procedures. It is a bee-shaped device consisting of two components: the body of the bee (vibration) and the removable ice wings (ice). The body of the bee consists of a vibrating motor powered by two alkaline AAA batteries. Vibration component can be activated by a switch located on the top part of the device. The ice wing component contains 18 g of ice and can be removed and kept in the freezer between procedures. Each pair of wings can stay frozen for about 10 min at room temperature and could be used up to 100 times.

Before the needle-related procedure, the wings are retrieved directly from the freezer of the unit as they should be solid frozen for optimal results. The wings are then inserted through elastic bands fixed on the back of the bee’s body. Afterwards, the Buzzy® device is placed by either attaching it to the arm or manually holding it in place, as close as possible above the needle insertion site (about 5 cm above the insertion site) and the vibration is activated. A 30 to 60 s interval is allotted between the installation of the device and the procedure. The device has to be kept in place throughout the procedure.

### How the intervention might work

The Buzzy® device is based on the gate control theory and the descending inhibitory controls. More specifically, the vibration is thought to block the afferent pain-receptive fibers (A-delta and C fibers) by the stimulation of the A-bêta non-noxious fibers which will activate an inhibitory interneuron resulting in reduction of the pain information transmitted to the spinal cord [23]. On the other hand, prolonged cold application (30–60 s) can stimulate the C nociceptive fibers and further blocks the A-delta pain transmission signal when applied close to the nociception source [24]. The stimulation of C fibers by cold application also transmits slow pain and noxious thermal information to the brain in activating a supraspinal modulation which increases the body’s overall pain threshold and therefore produces a generalized hypoalgesia at the insertion site [25, 26].

### Why it is important to do this review

Since the conception of the Buzzy device, studies have examined its effect on needle-related procedure. However, to our knowledge, no previous systematic reviews have been conducted to critically appraise evidence and the quality of these studies and potentially examine their combined effect. A systematic review is also needed to guide future research on this topic and for knowledge translation.

## Objectives

The overall aim of this systematic review is to examine the evidence for the efficacy of a device combining cold and vibration (Buzzy®) for needle-related procedural pain in children. More specifically, this review’s objectives are:

1. To systematically review trials using the Buzzy® device for pain management during needle-related procedures
2. To assess the impact of age on the efficacy of the Buzzy® device
3. To assess the risk of bias of the studies retained as well as the overall quality of the evidence for the main outcomes

## Methods

This systematic review protocol has been developed based on the Preferred Reporting Items for Systematic Reviews and Meta-Analyses Protocols (PRISMA-P) guidelines [27]. The protocol was registered with the PROSPERO database (Registration ID: CRD42017076531).

### Criteria for considering studies for this review

#### Type of studies

Eligible studies will be randomized controlled trials (RCTs), regardless of the language, date of publication, and publication status.

#### Type of participants

Studies involving infants, toddlers, children, and adolescents from 28 days to 18 years old requiring a needle-related procedure will be included. In this review, we define needle-related procedures as any procedure involving the use of needles for medical purposes, such as immunization, venipuncture, intravenous insertions, intramuscular, or subcutaneous injections.

#### Types of interventions

Only studies using simultaneously cold and vibration of the Buzzy® device will be included. Studies using only the vibration component of the Buzzy® device will be excluded as the effect resulting from the combination of both cold and vibration could be different from the effect of either one alone. We will include studies combining the Buzzy® device with other pharmacological or non-pharmacological/psychological interventions. However, if there is no data specifically on the effect of the Buzzy® device, we will conduct a sensitivity analyses excluding these studies where the Buzzy device is used with other co-interventions.

In addition, only studies with at least one control arm will be included. The comparator can be either no intervention, topical anesthetics, vapocoolant spray, non-pharmacological, or psychological interventions, or standard care as per the institution’s protocol.

#### Types of outcome measures

##### Primary outcomes

The primary outcome will be needle-related procedural pain as assessed by at least one of the following:

- Self-report pain scales (i.e., may include variations of Visual Analogue Scale (VAS), Numerical Rating Scales (NRS), Verbal Rating Scales (VRS), or face scales used to assess pain)
- Behavioral/observational pain scales (i.e., may include but are not limited to the Children’s Hospital of Eastern Ontario Pain Scales (CHEOPS) [28] and the Faces Legs Activity Cry Consolability Scale (FLACC) [29])

All pain scales must be validated for use in the population of interest. In this review, we define procedural pain intensity as the reported or observed pain level during the needle-related procedure evaluated either during or immediately after the procedure.

##### Secondary outcomes

The following secondary outcomes will be considered:

- Self-reported procedural anxiety (i.e., distress, fear, and/or stress) (may include variations of VAS, NRS, VRS, or face scales used to assess distress)
- Observed procedural anxiety (i.e., distress, fear, stress) (may include but are not limited to the Procedure Behavior CheckList (PCBL) [30], Observational Scale of Behavioral Distress (OSBD) [31], or variations of the Child-Adult Medical Procedure Interaction Scale (CAMPIS) [32–34])
- Success of the needle-related procedure for venipunctures and intravenous catheter insertions
- Healthcare professionals’ satisfaction and/or acceptability (e.g., satisfaction survey, questionnaires)
- Occurrence of side effects or adverse events
- Impact on diagnostic blood specimen collection results

### Search methods for identification of studies

#### Electronic searches

The search strategy for each database will be validated by a librarian information specialist familiar with the topic. The electronic search will be tailored for each database to include its specific keywords and MeSH terms. Searches from the following databases will be conducted to identify relevant randomized controlled trials (RCTs) published up to January 2018:

- Cochrane Central Register of Controlled Trials (CENTRAL)
- PubMed
- MEDLINE via Ovid
- EMBASE
- PsycInfo
- Web of Science
- Cumulative Index to Nursing and Allied Health Literature (CINAHL)

A draft of the search strategy and terms used for one of the databases is presented in Additional file 1.

#### Other sources of information

In addition to the electronic searches in the databases, we will review references and citation lists of included studies, as well as guidelines, reviews, and pediatric conference proceedings, and contact experts in the field to inquire about other potential studies not identified in the literature search. In addition, a search will be run through Google Scholar and ProQuest to make sure that no relevant studies were missed. The website (buzzyhelps.com) of the Buzzy® device will be consulted as it regularly publishes updates on new research. Finally, we will search for ongoing trials within the World Health Organization International Clinical Trials Registry Platform (WHO ICTRP), ClinicalTrials.gov (clinicaltrials.gov), and the metaRegister of controlled trials (mRCT) as they are the primary registries recognized by the ICMJE in order to identify unpublished and ongoing trials.

### Data collection and analysis

#### Selection of studies

All identified citations will be entered in a single Endnote library, and duplications will be removed. Two independent reviewers (AB, CK) will screen citations (titles and abstracts) in the Endnote library based on predefined eligibility criteria. Full-text articles will then be obtained and reviewed for citations considered relevant, potentially relevant, or with unclear relevance to the review by at least one reviewer. Full-text articles will be independently assessed by the same two reviewers (AB, CK) to determine whether or not they meet inclusion criteria. Selection discrepancies will be resolved by consensus or by consulting a third reviewer (SLM). Reasons for exclusion at this stage will be documented. Inter-rater reliability between the two reviewers extracting the data will be computed using a Kappa statistic. A flow diagram will be included to document all these steps according to the PRISMA guidelines [35].

#### Data extraction and management

Data extraction will be conducted independently by two reviewers (AB, CK) using a data extraction form developed for the purposes of this review. For each included study, reviewers will record relevant data about participant demographics, study setting(s), type of needle-related procedure, details on the intervention and the control arm, randomization techniques, outcomes, and other relevant variables. Any discrepancies will be resolve through discussion, or a third reviewer (SLM) will be consulted. The agreement among data extractors will be assessed for the primary outcomes data through the Kappa statistic.

#### Assessment of risk of bias in included studies

The risk of bias will be assessed using the Cochrane Collaboration Risk of Bias Tool [36]. This two-part tool covers seven specific domains of potential bias: sequence generation, allocation concealment, blinding of participants and personnel, blinding of outcome assessment, incomplete outcome data, and selective outcome reporting [36, 37]. The first part of the tool is achieved by describing what was reported in the study for each entry of the seven domains [36, 37]. The second part of the tool consists in the assignment of a judgment relating to the risk of bias for each entry, which could be categorized as “low risk” of bias, “high risk” of bias, or “unclear” risk of bias [36, 37]. Included studies will be assessed independently by two reviewers (AB, CK), and the results will be compared to reach a consensus. As suggested by the *Cochrane Handbook for Systematic Reviews of Interventions* [36], a study will be considered to have a “high risk” of bias if one of the evaluated domains is considered to have a “high risk” of bias. However, given the nature of the Buzzy® intervention which is almost not possible to blind, it is expected that most or all studies would present a “high risk” of bias on “blinding of participants and personnel” domain. Nonetheless, this will not affect the overall assessment of the study as “high risk” as the magnitude of bias associated with this lack of blinding is considerable and is likely to influence the primary outcome (pain intensity) given its subjective nature [36].

#### Measures of treatment effect

All statistical analysis will be performed using the Review Manager (RevMan) software (version 5.3. Copenhagen: The Nordic Cochrane Centre, The Cochrane Collaboration, 2014). For continuous outcomes, mean scores and standard deviations in experimental and control groups will be extracted. For dichotomous outcomes, the number of events for each group will be reported.

#### Unit of analysis issues

The unit of analysis will be the infants, children, and adolescents receiving either the Buzzy® device or a control intervention during a needle-related procedure. For studies using repeated measures design, we will only select pain and anxiety outcomes occurring during the needle-related procedure. If these outcomes were not assessed during the procedure, we will consider the next available measure closest in time to the procedure. If both measures are taken (i.e., during the procedure and immediately after), the measure during the procedure will be prioritized. For studies with multiple intervention groups, the primary comparison will be the Buzzy® device compared to the control arm instead of the other interventional arm. For a cross-over trial, we will treat the study as an RCT and therefore consider only the outcomes of the participants receiving the first intervention.

#### Dealing with missing data

If needed, the authors of identified studies will be contacted by email for missing data relevant for potential pooling (e.g., means, standard deviations, confidence intervals) or to obtain other missing details or clarifications. When authors cannot be contacted, we will make attempts to calculate other reported measures of variation by using the statistical formula, as recommended by the *Cochrane Handbook for Systematic Review for Intervention*. If there is insufficient data to perform these analyses or if the authors of the studies did not answer our query, the missing data will be excluded from this review.

#### Assessment of heterogeneity

For each outcome reporting the effect of two or more studies, statistical heterogeneity among studies will be assessed, before calculating a combined effect, using both chi-square statistic and *I*^2^. For the chi-square statistic, a *p* value of 0.10 to determine the statistical significance will be used instead of the conventional level of 0.05 as this test is known to have low statistical power in meta-analysis [38]. A statistically significant result indicates the presence of a problem of heterogeneity [38]. For the interpretation of the *I*^2^ statistic, we will follow the threshold established by the *Cochrane Handbook for Systematic Reviews for Interventions* (0–40%: might not be important; 30–50%: may represent moderate heterogeneity; 50–60%: may represent substantial heterogeneity; 75–100%: considerable heterogeneity) [38]. We will only conduct a meta-analysis if *I*^2^ is less than 50% [39]. If there is substantial heterogeneity (> 50%) or if the results of the chi-square is statistically significant, a narrative synthesis will be done instead of a meta-analysis.

#### Assessment of reporting bias

The publication bias and selective reporting of results will be assessed using funnel plot analyses of asymmetry [40] and the Egger’s test [41].

#### Data synthesis

If applicable, we will conduct a meta-analysis with a random effects model by pooling the data for the most comparable outcomes. Outcome data must be available from at least two studies to be considered for pooling. For continuous outcomes, we will calculate the standardized mean differences (SMDs) with a 95% confidence interval. For dichotomous outcomes, we will pool events between groups across studies using risk ratios (RRs) with a 95% confidence interval. If a meta-analysis is not feasible, we will provide a narrative review of the findings.

#### Quality of evidence

The quality of evidence across studies for each outcome will be assessed using the Grades of Recommendation, Assessment, Development and Evaluation (GRADE) system via the GRADE profiler Guideline Development Tool software (2015, McMaster University and Evidence Prime Inc.). As proposed by the GRADE working group [42] and the *Cochrane Handbook for Systematic Reviews for Interventions* [43], we will consider five domains to evaluate the certainty of evidence: risk of bias, inconsistency, indirectness, imprecision, and publication bias. We will use four categories of evidence of quality based on the overall GRADE scores (high = 4 points, moderate = 3 points, low = 2 points, very low = 1 point or less). As all the included studies will be randomized controlled trials, the initial GRADE score of each outcome will be set at “high” (4 points), then it could possibly be downgraded depending on the evaluation of the remaining four domains. The decision to decrease the grade will be supported by the criteria for assessing the grade of evidence, as established by the GRADE working group [42]. Results will be presented in a summary of findings table.

#### Subgroup analysis

If a meta-analysis is carried out, subgroup analyses will be performed regardless of the amount of heterogeneity using a random effects model. A chi-square test for subgroup differences as well as the *I*^2^ statistic will be computed and reported for each subanalysis.

1. Age: Included studies will be categorized by age groups according to the Eunice Kennedy Shriver National Institute of Child Health and Human Development (NICHD) Pediatric Terminology cited by Williams et al. [44]: infancy and toddler, 28 days to < 2 years old; early childhood, 2–5 years old; middle childhood, 6–11 years old; adolescence, 12–18 years old. We will use the same method used by Birnie et al. [45] for the categorization of studies into subgroups based on the overall mean or median age of the study population. When a study reports the results separately for each age group, these age groups will be considered separately in the age subgroup analysis rather than the overall study results.
2. Types of needle-related procedures: If applicable, subgroup analyses will be carried out according to the type of needle-related procedure (e.g., IV catheter insertion, venipuncture, intramuscular injection, subcutaneous injection, heel lance).
3. Risk of bias: Subgroup analyses will examine the effect of low vs. high or unclear risk of bias.

## Discussion

To our knowledge, this is the first systematic review assessing the combining effects of the Buzzy® device for needle-related pain management in children. Results will provide a detailed summary of evidence regarding its use in children undergoing a needle-related procedure. Strengths and limitations of included studies and this review will be discussed, and recommendations for further research and clinical practice will be provided.

## Additional file

Additional file 1: Draft of the search strategy and terms used for one of the databases. (DOCX 133 kb)

## Abbreviations

CINAHL: Cumulative Index to Nursing and Allied Health Literature
EMBASE: Excerpta Medica database
GRADE: Grades of Recommendation, Assessment, Development and Evaluation
ICMJE: International Committee of Medical Journal Editors
MEDLINE: Medical Literature Analysis and Retrieval System Online
MeSH: Medical Subject Headings
mRCT: metaRegister of controlled tirals
PRISMA: Preferred Reporting Items for Systematic Reviews and Meta-analysis
PRISMA-P: Preferred Reporting Items for Systematic Reviews and Meta-analysis Protocols
RCT: Randomized controlled trial
RRs: Risk ratios
SMDs: Standardized mean differences
WHO ICTRP: World Health Organization International Clinical Trials Registry Platform
