# Supplementary material for: Efficacy of the Buzzy® device for pain management of children during needle-related procedures: a systematic review protocol
Source: Syst Rev. 2018 May 22;7:78. doi: 10.1186/s13643-018-0738-1 (PMC5964660; doi:10.1186/s13643-018-0738-1)
Supplement: Supplementary file 1 — Draft of the search strategy and terms used for one of the databases. (DOCX 133 kb) [file 13643_2018_738_MOESM1_ESM.docx]

**Additional file**

Draft of search strategy for one electronic database

## PubMed

#1 (Vibration[mh] AND (Cold Temperature[mh] OR Cryotherapy[mh:noexp]))

#2 ((cooling[tiab] OR cool[tiab] OR cold[tiab] OR coldness[tiab] OR refriger*[tiab] OR cryot*[tiab]) AND vibrati*[tiab]) OR ((cooling[OT] OR cool[OT] OR cold[OT] OR coldness[OT] OR refriger*[OT] OR cryot*[OT]) AND vibrati*[OT]) OR Buzzy*[tiab] OR Buzzy*[OT]

#3 Pain[mh] OR Pain management[mh] OR Pain Measurement[mh] OR Anxiety[mh:noexp] OR fear[mh]

#4 Discomfort*[tiab] OR Pain*[tiab] OR Anx*[tiab] OR fear*[tiab] OR distress*[tiab] OR Discomfort*[OT] OR Pain*[OT] OR Anx*[OT] OR fear*[OT] OR distress*[OT]

#5 Blood Specimen Collection[mh] OR Administration, Intravenous[mh] OR Injections[mh] OR Needles[mh] OR Immunization[mh]

#6 Venipuncture*[tiab] OR Venepuncture*[tiab] OR Venesection*[tiab] OR Venisection*[tiab] OR Needle*[tiab] OR IV[tiab] OR Intravenous[tiab] OR Intra venous[tiab] OR Phlebotom*[tiab] OR Injection*[tiab] OR Injectable*[tiab] OR Insertion*[tiab] OR vascular access*[tiab] OR cannula*[tiab] OR immuni*[tiab] OR inoculat*[tiab] OR vaccin*[tiab] OR subcutan*[tiab] OR puncture*[tiab] OR catheter*[tiab] OR heel lanc*[tiab] OR finger prick*[tiab] OR heel prick*[tiab] OR heel stick*[tiab] OR sutur*[tiab] OR aspiration[tiab] OR spinal tap*[tiab] OR biops*[tiab] OR port-a-cath*[tiab] OR portacath*[tiab] OR (line[tiab] AND (insert*[tiab] or remov*[tiab])) OR ((Blood[tiab] OR specimen[tiab]) AND (Draw*[tiab] OR collect*[tiab] OR sampl*[tiab])) OR venipuncture*[OT] OR Venepuncture*[OT] OR Venesection*[OT] OR Venisection*[OT] OR Needle*[OT] OR IV[OT] OR Intravenous[OT] OR Intra venous[OT] OR Phlebotom*[OT] OR Injection*[OT] OR Injectable*[OT] OR Insertion*[OT] OR vascular access*[OT] OR cannula*[OT] OR immuni*[OT] OR inoculat*[OT] OR vaccin*[OT] OR subcutan*[OT] OR puncture*[OT] OR catheter*[OT] OR heel lanc*[OT] OR finger prick*[OT] OR heel prick*[OT] OR heel stick*[OT] OR sutur*[OT] OR aspiration[OT] OR spinal tap*[OT] OR biops*[OT] OR port-a-cath*[OT] OR portacath*[OT] OR (line[OT] AND (insert*[OT] or remov*[OT])) OR ((Blood[OT] OR specimen[OT]) AND (Draw*[OT] OR collect*[OT] OR sampl*[OT]))

#7 (#1 OR #2) AND (#3 OR #4) AND (#5 OR #6)

#8 Infant[MH] OR Child[MH] OR Adolescent[MH] OR Intensive Care, Neonatal[MH] OR Intensive Care Units, Neonatal[MH] OR Intensive Care Units, Pediatric[MH] OR Hospitals, Pediatric[MH] OR Neonatology[MH] OR Neonatal Nursing[MH] OR Nurses, Pediatric[MH] OR Nurseries[MH] OR Perinatology[MH] OR Perinatal Care[MH] OR Pediatrics[MH] OR Pediatricians[MH] OR Child, Hospitalized[MH] OR Child, Institutionalized[MH] OR Adolescent, Hospitalized[MH] OR Adolescent, Institutionalized[MH] OR newborn*[tw] OR new born*[tw] OR babie*[tw] OR baby*[tw] OR infant*[tw] OR infancy[tw] OR toddler*[tw] OR preschool*[tw] OR pre school*[tw] OR child*[tw] OR kid[tw] OR kid'[tw] OR kids[tw] OR kid's[tw] OR boy[tw] OR boy'[tw] OR boys[tw] OR boy's[tw] OR girl[tw] OR girl'[tw] OR girls[tw] OR girl's[tw] OR schoolchild*[tw] OR juvenil*[tw] OR preadolescen*[tw] OR youth*[tw] OR adolescen*[tw] OR teen*[tw] OR puber[tw] OR puber'[tw] OR pubers[tw] OR puber's[tw] OR pubert*[tw] OR pubescen*[tw] OR high school*[tw] OR highschool*[tw] OR secondary school*[tw] OR paediatric*[tw] OR pediatric*[tw] OR PICU*[tw] OR neonat*[tw] OR neo nat*[tw] OR NICU*[tw]

#9 #7 AND #8

((Vibration[mh] AND (Cold Temperature[mh] OR Cryotherapy[mh:noexp])) OR ((cooling[tiab] OR cool[tiab] OR cold[tiab] OR coldness[tiab] OR refriger*[tiab] OR cryot*[tiab]) AND vibrati*[tiab]) OR ((cooling[OT] OR cool[OT] OR cold[OT] OR coldness[OT] OR refriger*[OT] OR cryot*[OT]) AND vibrati*[OT]) OR Buzzy*[tiab] OR Buzzy*[OT]) AND (Pain[mh] OR Pain management[mh] OR Pain Measurement[mh] OR Anxiety[mh:noexp] OR fear[mh] OR Discomfort*[tiab] OR Pain*[tiab] OR Anx*[tiab] OR fear*[tiab] OR distress*[tiab] OR Discomfort*[OT] OR Pain*[OT] OR Anx*[OT] OR fear*[OT] OR distress*[OT]) AND (Blood Specimen Collection[mh] OR Administration, Intravenous[mh] OR Injections[mh] OR Needles[mh] OR Immunization[mh] OR Venipuncture*[tiab] OR Venepuncture*[tiab] OR Venesection*[tiab] OR Venisection*[tiab] OR Needle*[tiab] OR IV[tiab] OR Intravenous[tiab] OR Intra venous[tiab] OR Phlebotom*[tiab] OR Injection*[tiab] OR Injectable*[tiab] OR Insertion*[tiab] OR vascular access*[tiab] OR cannula*[tiab] OR immuni*[tiab] OR inoculat*[tiab] OR vaccin*[tiab] OR subcutan*[tiab] OR puncture*[tiab] OR catheter*[tiab] OR heel lanc*[tiab] OR finger prick*[tiab] OR heel prick*[tiab] OR heel stick*[tiab] OR sutur*[tiab] OR aspiration[tiab] OR spinal tap*[tiab] OR biops*[tiab] OR port-a-cath*[tiab] OR portacath*[tiab] OR (line[tiab] AND (insert*[tiab] or remov*[tiab])) OR ((Blood[tiab] OR specimen[tiab]) AND (Draw*[tiab] OR collect*[tiab] OR sampl*[tiab])) OR venipuncture*[OT] OR Venepuncture*[OT] OR Venesection*[OT] OR Venisection*[OT] OR Needle*[OT] OR IV[OT] OR Intravenous[OT] OR Intra venous[OT] OR Phlebotom*[OT] OR Injection*[OT] OR Injectable*[OT] OR Insertion*[OT] OR vascular access*[OT] OR cannula*[OT] OR immuni*[OT] OR inoculat*[OT] OR vaccin*[OT] OR subcutan*[OT] OR puncture*[OT] OR catheter*[OT] OR heel lanc*[OT] OR finger prick*[OT] OR heel prick*[OT] OR heel stick*[OT] OR sutur*[OT] OR aspiration[OT] OR spinal tap*[OT] OR biops*[OT] OR port-a-cath*[OT] OR portacath*[OT] OR (line[OT] AND (insert*[OT] or remov*[OT])) OR ((Blood[OT] OR specimen[OT]) AND (Draw*[OT] OR collect*[OT] OR sampl*[OT])) AND (Infant[MH] OR Child[MH] OR Adolescent[MH] OR Intensive Care, Neonatal[MH] OR Intensive Care Units, Neonatal[MH] OR Intensive Care Units, Pediatric[MH] OR Hospitals, Pediatric[MH] OR Neonatology[MH] OR Neonatal Nursing[MH] OR Nurses, Pediatric[MH] OR Nurseries[MH] OR Perinatology[MH] OR Perinatal Care[MH] OR Pediatrics[MH] OR Pediatricians[MH] OR Child, Hospitalized[MH] OR Child, Institutionalized[MH] OR Adolescent, Hospitalized[MH] OR Adolescent, Institutionalized[MH] OR newborn*[tw] OR new born*[tw] OR babie*[tw] OR baby*[tw] OR infant*[tw] OR infancy[tw] OR toddler*[tw] OR preschool*[tw] OR pre school*[tw] OR child*[tw] OR kid[tw] OR kid'[tw] OR kids[tw] OR kid's[tw] OR boy[tw] OR boy'[tw] OR boys[tw] OR boy's[tw] OR girl[tw] OR girl'[tw] OR girls[tw] OR girl's[tw] OR schoolchild*[tw] OR juvenil*[tw] OR preadolescen*[tw] OR youth*[tw] OR adolescen*[tw] OR teen*[tw] OR puber[tw] OR puber'[tw] OR pubers[tw] OR puber's[tw] OR pubert*[tw] OR pubescen*[tw] OR high school*[tw] OR highschool*[tw] OR secondary school*[tw] OR paediatric*[tw] OR pediatric*[tw] OR PICU*[tw] OR neonat*[tw] OR neo nat*[tw] OR NICU*[tw]))
